# Supplementary material for: The use of heuristics in genetic testing decision-making: A qualitative interview study
Source: PLoS One. 2021 Nov 30;16(11):e0260597. doi: 10.1371/journal.pone.0260597 (PMC8631642; doi:10.1371/journal.pone.0260597)
Supplement: S1 File — (DOCX) [file pone.0260597.s001.docx]

**Supporting information file S1: Author details and COREQ checklist**

1. **Author details**

Bettina Maria Zimmermann (BMZ), lead author, BA, MSc, female was a PhD student at the Institute for Biomedical Ethics at the University of Basel when the study was conducted. The project is part of her dissertation. BZ has an educational background in social sciences and medical genetics and has been trained as part of her PhD in qualitative methods. With her background in medical genetics, BZ has a generally positive attitude towards genetic testing and technological progress in general. She kept reflective diary while conducting and analysing interviews and had regular reflective exchange sessions with IK (who has a more skeptical attitude towards genetic testing in the clinical setting).

David Martin Shaw (DMS), Dr. phil., MA, MSc, MML, male, is a senior researcher at the Institute for Biomedical Ethics at the University of Basel and BZ' PhD co-supervisor. DS has a background in philosophy and is experienced in both conceptual and empirical work in the field of bioethics. He has published a number of articles on tethical issues related to genetics, and is also Assistant Professor at the Care and Public Health Research Institute at Maastricht University.

Bernice Elger (BE), Prof. Dr. med., female, is a professor at the University of Basel and head of the Institute for Biomedical Ethics. She is BZ' PhD co-supervisor and has a background in medicine and theology. She is an experienced researcher in the field of empirical bioethics and has habilitated in genetic testing for Huntington's disease.

Insa Koné (IK), Dr. med., female, is a postdoctoral researcher at the Institute for Biomedical Ethics at the University of Basel. She is also working in clinical practice as a general practitioner and is experienced in both quantitative and qualitative research.

1. **COREQ (COnsolidated criteria for REporting Qualitative research) Checklist**

| **Topic** | **Item No.** | **Guide Questions/Description** | **Reported on Page No.** |
| --- | --- | --- | --- |
| **Domain 1: Research team and reflexivity** |  |  |  |
| *Personal characteristics* | | |  |
| Interviewer/facilitator | 1 | Which author/s conducted the interview or focus group? | 8 |
| Credentials | 2 | What were the researcher’s credentials? E.g. PhD, MD | S1 |
| Occupation | 3 | What was their occupation at the time of the study? | S1 |
| Gender | 4 | Was the researcher male or female? | S1 |
| Experience and training | 5 | What experience or training did the researcher have? | S1 |
| *Relationship with participants* |  |  |  |
| Relationship established | 6 | Was a relationship established prior to study commencement? | 8 |
| Participant knowledge of the interviewer | 7 | What did the participants know about the researcher? e.g. personal goals, reasons for doing the research | 8 |
| Interviewer characteristics | 8 | What characteristics were reported about the inter viewer/facilitator? e.g. Bias, assumptions, reasons and interests in the research topic | S1 |
| **Domain 2: Study design** | | |  |
| *Theoretical framework* |  |  |  |
| Methodological orientation and Theory | 9 | What methodological orientation was stated to underpin the study? e.g.  grounded theory, discourse analysis, ethnography, phenomenology, content analysis | 7, 9 |
| *Participant selection* |  |  |  |
| Sampling | 10 | How were participants selected? e.g. purposive, convenience, consecutive, snowball | 7-8 |
| Method of approach | 11 | How were participants approached? e.g. face-to-face, telephone, mail, email | 7 |
| Sample size | 12 | How many participants were in the study? | 10 |
| Non-participation | 13 | How many people refused to participate or dropped out? Reasons? | 7 |
| *Setting* |  |  |  |
| Setting of data collection | 14 | Where was the data collected? e.g. home, clinic, workplace | 8 |
| Presence of nonparticipants | 15 | Was anyone else present besides the participants and researchers? | 8 |
| Description of sample | 16 | What are the important characteristics of the sample? e.g. demographic data, date | 10 (Table 1) |
| *Data collection* |  |  |  |
| Interview guide | 17 | Were questions, prompts, guides provided by the authors? Was it pilot tested? | 8 |
| Repeat interviews | 18 | Were repeat interviews carried out? If yes, how many? | n.a |
| Audio/visual recording | 19 | Did the research use audio or visual recording to collect the data? | 9 |
| Field notes | 20 | Were field notes made during and/or after the interview or focus group? | 8 |
| Duration | 21 | What was the duration of the interviews or focus group? | 10 |
| Data saturation | 22 | Was data saturation discussed? | 8 |
| Transcripts returned | 23 | Were transcripts returned to participants for comment and/or correction? | 9 |
| **Domain 3: analysis and findings** | | |  |
| *Data analysis* |  |  |  |
| Number of data coders | 24 | How many data coders coded the data? | 9 |
| Description of the coding tree | 25 | Did authors provide a description of the coding tree? | File S4 |
| Derivation of themes | 26 | Were themes identified in advance or derived from the data? | 9 |
| Software | 27 | What software, if applicable, was used to manage the data? | 9 |
| Participant checking | 28 | Did participants provide feedback on the findings? | 9 |
| *Reporting* |  |  |  |
| Quotations presented | 29 | Were participant quotations presented to illustrate the themes/findings?  Was each quotation identified? e.g. participant number | 10-19 |
| Data and findings consistent | 30 | Was there consistency between the data presented and the findings? | 10-19 |
| Clarity of major themes | 31 | Were major themes clearly presented in the findings? | 10-19 |
| Clarity of minor themes | 32 | Is there a description of diverse cases or discussion of minor themes? | 10-19 |

Developed from: Tong A, Sainsbury P, Craig J. Consolidated criteria for reporting qualitative research (COREQ): a 32-item checklist for interviews and focus groups. *International Journal for Quality in Health Care*. 2007. Volume 19, Number 6: pp. 349 – 357
