# Supplementary material for: The use of heuristics in genetic testing decision-making: A qualitative interview study
Source: PLoS One. 2021 Nov 30;16(11):e0260597. doi: 10.1371/journal.pone.0260597 (PMC8631642; doi:10.1371/journal.pone.0260597)
Supplement: S2 File — (DOCX) [file pone.0260597.s002.docx]

# Supporting information file S2

## Interview guide patient interviews

*Please note: the interview guide was adapted in the course of the study according to findings. Vignettes were introduced after six interviews. Vignette 2 was removed from the interview guide after interview 11. Question 7 was introduced after interview 11. The other changes concerned the follow-up questions that were asked depending on the participant’s reactions to the main questions (bold).*

1. **When did you first get in touch with genetic testing? Why did you go to genetic counselling?**
2. **How was your decision-making process regarding genetic testing?**
   - What were the reasons for your decision?
   - Which factors influenced your decision?
   - Who did you exchange your thoughts with during the decision-making process?
   - Was your decision more of a gut or a head decision?
3. **How did you feel during the testing process?**
   - Who were your exchange partners during this time?
   - How did you deal with the uncertainty?
4. **Do you experience or fear positive or negative consequences due to your genetic test?**
   - If so, what are these consequences?
   - How do you deal with the test result?
   - How do you deal with the consequences? Do you have specific strategies?
5. **With whom do you talk about genetic testing and its consequences?**
   - Have other family members also had themselves tested?
   - Who can or do you not want to talk to about it? Why?
   - Can you talk openly about the subject?
6. **If an at-risk individual would ask for your advice regarding genetic testing, what would you reply?**
7. **Do you associate something negative/positive with genetic testing?**

*(ask for an opposite position than previously displayed by the participant)*

1. **Where did you find information about genetic testing?**
   - What were your expectations of the genetic consultations?
   - Did you obtain information about the genetic test from other sources in addition to genetic counselling? If so, where? What kind of information?
   - Would you have liked to have more alternative sources of information?
   - Did you hear/read about genetic testing in the media?
   - What do you think about the presentation of genetic tests in the media?
   - What were your information needs? What interested you most?
2. **Is there anything else on the subject that you would like to add?**
3. **Finally, may I ask you for some demographic information?**
   - How old are you?
   - What is your profession? What is your education?
   - If not already mentioned: What is your civil status? Do you have siblings? Do you have children?
   - Do you live in the city or in the countryside?

Hypothetical vignettes:

**Vignette 1**

Imagine there is a genetic test that can determine an increased risk of 40% for a possible outbreak of a severe, fatal hereditary disease. The exact time of onset of the disease cannot be determined. Unfortunately, there are no preventive measures for this disease and treatment is only possible symptomatically (to alleviate suffering). You have already lost one parent to this disease.

- Would you want to do a genetic test or not? Why (not)?
- What if the risk only increased by 10-20%? Or by 50-80%? Would there be a percentage threshold for you as to when the test is still useful?
- What if the outbreak could be predicted to be 100%?

**Vignette 2**

Imagine a friend of yours should undergo a genetic test. If the test result is positive, the risk of getting the disease would rise to 40% and there would be six-monthly check-ups to detect the disease as early as possible. Your friend, however, has been planning a two-year trip around the world for a long time, and the preventive examinations would not be feasible during this period. In your opinion, should your friend have the genetic test anyway?

**Vignette 3**

Imagine your family doctor telling you about a genetic test that can tell you how high your personal genetic risk is of contracting a widespread disease - for example, diabetes, high blood pressure or a heart attack. This risk is also influenced by personal behaviours, such as diet, exercise, etc. Would you like to take such a test? Who would you recommend this test to? What are the advantages and disadvantages of this test?
