# Supplementary material for: The use of heuristics in genetic testing decision-making: A qualitative interview study
Source: PLoS One. 2021 Nov 30;16(11):e0260597. doi: 10.1371/journal.pone.0260597 (PMC8631642; doi:10.1371/journal.pone.0260597)
Supplement: S3 File — (DOCX) [file pone.0260597.s003.docx]

# Supporting information file S3

## Interview guide expert interviews

Thank you for taking the time and speaking with me! To complement the patient interviews, I would like to ask you, as an expert, some additional questions that arose during the patient interviews. This interview will take approximately 60 minutes and is confidential. Only the analyzing team and I have direct access to the tape. All the mentioned names and places will be encrypted in the transcript. In publications, care is taken to ensure that no statements can be traced back to your institution (and thus indirectly to you as a person) unless you explicitly agree. Some questions are rather technical, but I am also interested in your personal opinion regarding improvement suggestions or problems.

Do you agree to this interview? Do you have any remaining questions before we start?

1. **We found that certainty and uncertainty play an important role in genetic testing. Do you remember the last genetic genetic consultation where this was discussed?**

a. What uncertainties do you face?

b. How do you convey certainty?

1. **From your perspective, how do patients cope with the information provided during the genetic consultation?**
   1. Do you experience some patients being overwhelmed?
   2. How do you assess the understanding of your patients after the consultations regarding risk assessment?
   3. When you are under the impression that patients do not fully understand the concept of risk, how do you deal with it?
   4. Do patients frequently ask for recommendations? How do you handle them?
2. **How often do you experience that patients are still undecided whether to take the test or not when coming to the first genetic consultation?**
3. **What concerns do patients address during the genetic consultation? Have patients ever mentioned to you that they find it difficult to talk to others about it?**
   1. With whom?
   2. What do you advise your patients to do in this case?

***Are there patients concerned about discrimination?***

1. What do you tell these patients?
2. How important do you think is the risk of discrimination in the case of diagnosed HBOC or Lynch Syndrome?
3. What kind of discrimination have you experienced with your patients?
4. **Do you remember patients who regretted their decision after testing?**
   1. Why?
   2. How did you and these patients cope with this situation?
   3. Does such an incident influence future genetic consultations?
5. In your opinion, who should be responsible for genetic counselling?
6. How is the information dissemination to family members handled?
7. Is it common to conduct BRCA genetic testing in cancer patients without genetic counselling? How does the cooperation between oncologists and geneticists look like?
8. Do you see patients that were counselled by medical doctors not specialized in genetics? How do you handle these situations?
9. How standardized is the referral procedure? Where do you see room for improvement?
10. What is the indication for panel testing in the case of HBOC?
11. Are there any statistics on how many people make use of genetic counselling and how many then decide not to take a test? If not: what do your estimate?
12. How often do you get questions from patients after the consultations?
13. Which information aids do you use in your genetic consultations?
14. Do individuals who have been tested positive perceive themselves as patients or as healthy individuals in the case of predictive testing? What is your impression?
15. What do you think about predictive genetic testing for nonactionable diseases?
16. What do you think about predictive genetic testing for diseases with high environmental influences, for instance for cardiovascular diseases or diabetes?
