# Supplementary material for: The use of heuristics in genetic testing decision-making: A qualitative interview study
Source: PLoS One. 2021 Nov 30;16(11):e0260597. doi: 10.1371/journal.pone.0260597 (PMC8631642; doi:10.1371/journal.pone.0260597)
Supplement: S4 File — (DOCX) [file pone.0260597.s004.docx]

**Supporting information file S4: Coding framework**

| **Code** | **Memo** |
| --- | --- |
| **1 Experiencing complexity** | Establishment / recognition of the complexity of the decision regarding genetic testing.  <-> Opposite: decision is simple |
| 1.1 The inevitable necessity of making a decision |  |
| Fate |  |
| No choice if one learns about the option |  |
| 1.2 Individual variance in perception of complexity |  |
| Decision not perceived as complex |  |
| Complexity to make a decision |  |
| 1.3 Emotional involvement |  |
| Variant vs mutation |  |
| Emotional reasons for and against testing |  |
| **2 Anticipating the test result** | Subjective assessment of the probability of being tested positive |
| Anticipation of test result supports decision + coping |  |
| Expected possibility to be positive |  |
| **3 Focusing on consequences** | The complexity of the decision reduced to presence or absence of medical consequences |
| One should deal with the consequences before testing |  |
| Consequence as an imperative in case of positive test |  |
| Consequences clear from the beginning |  |
| Aim to avoid becoming sick |  |
| Consequences discussed during counseling |  |
| **4 Dealing with information** | Avoidance vs active gathering of information  Postponing the decision of whether to take the test (age-related) |
| Reluctance to learn details about test result |  |
| Postponing the test |  |
| Gathering information |  |
| **5 Interpreting disease risk** | Risk is difficult for people to conceptualize (see Lit. Psychology). How do the participants deal with it? What auxiliary constructs do they have?  Complexity reduction by orientation to others: Needs of untested family members, decision of tested family members, recommendation of doctors. What happens when you don't "go with the mainstream"? |
| Longing for 100% certainty |  |
| Being sick or being healthy |  |
| Picturing risks in black and white |  |
| Quantifying disease risk |  |
| **6 Using external guidance** |  |
| Testing for children |  |
| Family influence |  |
| Relying on other people affected |  |
| Relying on healthcare professionals |  |
| Health insurance coverage |  |
| Reluctance to talk to others |  |
| **7 (Re-)Considering the general uncertainty of life** | Dealing with genetic risk by seeing it relative to other health risks. |
