# Supplementary material for: The use of heuristics in genetic testing decision-making: A qualitative interview study
Source: PLoS One. 2021 Nov 30;16(11):e0260597. doi: 10.1371/journal.pone.0260597 (PMC8631642; doi:10.1371/journal.pone.0260597)
Supplement: Attachment — Submitted filename: Response to reviewers.docx [file pone.0260597.s006.docx]

# Response to reviewers

Reviewer #2: The comments were well answered. The paper is interesting and it is good to be able to publish and reflect on well explained qualitative research. The choice of "minor revisions" concerns a few points of English that are a bit awkward and a few typos. It's not a big thing and an additional proofreading by a native English speaker could improve the flow of lecture.

Author response: We thank reviewer 2 for their positive response. An English native speaker with a degree in English language has revised the language of the paper.

Reviewer #3: (No Response)

Reviewer #4: 1. The justification for two additional interviewees (experts) remains unclear. Although the authors have included the interview guide, expert quotes are not included in the results or highlighted in the discussion. The analytic approach to the expert interviews is not clearly reported.
Author response: We thank reviewer 4 for their comments and clarifications. We highlighted the analytic approach in the methods section (page 10), it reads: “We conducted these interviews after an initial analysis of all counselee interviews and included them in the analysis to examine whether experts supported or contradicted or general understanding of interview data.” It also reads in the first section of findings: “Expert interviews were used to confirm and contrast counselees' views.” Along with this analytic approach, we repeatedly highlighted in the results where experts supported or contrasted our findings. For example, on page 13 it reads: “Conversely, one expert reflected on the difficulty of explaining these variants to counselees in the context of panel testing, stating that those lower risks or variants of unknown significance added complexity that was difficult to communicate. This expert feared that counselees would instead overestimate the risks of these variants.”
While we do indirectly cite experts in the results, we chose not to select direct quotes from experts. When selecting illustrative quotes, we attempted to illustrate our findings while maintaining a readable structure to the readers. We think that the quotes from counselees serve this purpose better.
The reason why expert views are not highlighted in the discussion is because they mainly supported our findings. This was also why we stopped recruiting experts after two interviews. We did consider where we could include expert views in the discussion, but decided that it was beyond the scope to do this more prominently because we did not ask them directly about group A and B heuristics.

2. My previous comment regarding a characterization in variation in preferences was not intended to suggest that the authors apply a quantitative approach to the analysis by reporting proportions. Rather, it would be helpful to provide a descriptive understanding of where opinions varied (or were consistent) across participants.
Author response: We thank reviewer 4 for the clarification. Indeed, our whole analytic approach aimed at examining variations and consistencies among counselees and experts. We report consistencies through the topics (heuristics) that were commonly reported, and then describe in detail the various views reported by participants. We clarified this in the first paragraph of the findings section (page 10).

3. Exclusion of pregnant people remains unclear. If this was an issue for ethical approval, please estate that in the paper.
Author response: It was indeed an issue of the ethics committee to not include vulnerable people, including pregnant women. We rephrased in the methods section, it now reads on page 8: “Counselees between the age of 18-70 that were not considered vulnerable due to mental state or pregnancy by the ethics committees that approved the study were eligible.»

4. The authors appear to have uploaded multiple versions of the appending documents (interview guides)
Author response: This is because we used different interview guides for patient and experts – File S2 is the interview guide used for patients, File S3 is the interview guide used for expert interviews.
